# Convergent subgenome dominance but with lineage-specific functional divergence of homoeologs during cave adaptation: insights from full-length transcriptomes of *Sinocyclocheilus* species

**DOI:** 10.1186/s12983-025-00591-1

**Published:** 2025-11-27

**Authors:** Shaohua Xu, Mingming Zhang, Fanwei Meng, Chongnv Wang, Xinxin Li, Baocheng Guo

**Affiliations:** 1 State Key Laboratory of Animal Biodiversity Conservation and Integrated Pest Management, CAS Key Laboratory of Animal Biodiversity Conservation and Integrated Pest Management, CAS Key Laboratory of Zoological Systematics and Evolution, Institute of Zoology, Chinese Academy of Sciences, Beijing, 100101 China; 2 University of Chinese Academy of Sciences, Beijing, 100049 China

**Keywords:** *Sinocyclocheilus*, Cavefish, Full-length transcriptome, Polyploid evolution, Extreme environment

## Abstract

Allopolyploidy creates duplicated genomes that drives evolutionary innovation and adaptive diversification under extreme environmental pressures. Although subgenomic architecture is recognized as pivotal in post-polyploid evolution, the mechanisms by which divergent subgenome dynamics shape adaptive potential remain unclear. We investigated how subgenome evolution relates to environmental adaptation in *Sinocyclocheilus* cavefish, an allotetraploid lineage that repeatedly colonized caves across the karst landscapes of Southwest China. We integrated full-length and short-read transcriptomes from a surface-dwelling species (*S. angustiporus*) and two independently cave-adapted species (*S. microphthalmus* and *S. furcodorsalis*). The two cave dweller species showed consistent B-subgenome dominance in homoeolog expression, whereas the surface species showed balanced expression. Functional enrichment analyses identified lineage-specific functional divergence of dominantly expressed homoeologs. In *S. microphthalmus*, subgenome-dominant genes were significantly enriched in immunological elements, suggesting an evolutionary shift in immune investment. In *S. furcodorsalis*, subgenome-dominant genes were significantly enriched in neuromodulatory and metabolic pathways, consistent with energy conservation and sensory regression in nutrient-poor caves. Together, these findings suggest that polyploidy can promote diversification through convergent subgenome dominance. Biased repurposing of distinct stress-responsive modules resolves ancestral genomic conflicts. This, in turn, drives the emergence of lineage-specific functions and links genome duplication to ecological adaptation.

**Supplementary Information:**

The online version contains supplementary material available at 10.1186/s12983-025-00591-1.

## Introduction

Polyploidization constitutes a major evolutionary force, generating genomic plasticity that enhances adaptive potential under extreme environmental conditions [1–3]. While recurrent polyploidy is widespread among angiosperms, the mechanisms by which genome duplication drives environmental specialization, particularly in harsh and isolated ecosystems, remain a fundamental question in evolutionary genomics [1]. Cave environments, characterized by perpetual darkness and nutrient scarcity, provide powerful natural laboratories for exploring how polyploid genomic architectures influence adaptive trajectories through subgenome evolution [4]. Cave-adapted organisms, spanning a broad taxonomic range from invertebrates to mammals, have emerged as critical models for dissecting the genomic basis of adaptation to extreme environments [5–7].

The cyprinid genus *Sinocyclocheilus*, comprising 83 recognized species [8–13], represents the most species-rich radiation of freshwater cavefish known to date [14]. Members of this genus exhibit remarkable morphological diversity, spanning surface-dwelling forms with fully developed eyes and pigmentation to cave-adapted phenotypes characterized by eye and scale degeneration, reduced or absent pigmentation, and extensive sensory system remodeling [15–19]. Distinct from most cavefish models, such as *Astyanax mexicanus*, *Sinocyclocheilus* underwent a recent shared allotetraploidization event with common carp and goldfish [20, 21], resulting in a composite genome formed from two divergent cyprinid progenitors before colonization of the karstic landscapes of Southwest China [22]. This allotetraploid architecture establishes *Sinocyclocheilus* as a unique vertebrate system for exploring the evolutionary consequences of genome duplication in extreme environments [23]. Its taxonomic richness, diverse phenotypic adaptations, and duplicated genome structure make the genus an excellent multi-species model for investigating the functional interplay between polyploidy and extreme ecological specialization [18, 24, 25].

To elucidate the regulatory architecture underlying adaptation to extreme subterranean environments, a subgenome-resolved transcriptomic comparison was conducted across three *Sinocyclocheilus* species, including the surface-dwelling *S. angustiporus* and the cave-dwelling *S. microphthalmus* and *S. furcodorsalis*, which represent phylogenetically divergent lineages exhibiting convergent adaptation to cave environments [8]. Full-length transcriptomes generated via PacBio Iso-Seq and Illumina short-read sequencing enabled the construction of high-confidence subgenome-resolved homoeolog annotations and quantitative expression profiles. Analysis revealed consistent B-subgenome dominance (SubB-dominance) in the phylogenetically distinct cave-dwelling species, in sharp contrast to the balanced subgenome dynamics observed in the surface relative. This convergent regulatory bias was associated with lineage-specific functional divergence, characterized by the subgenomic repurposing of immune-related modules in *S. microphthalmus* and neuromodulatory and metabolic pathways in *S. furcodorsalis*. These findings suggest that polyploidy drives adaptation to cave environments through convergent subgenome dominance. In this process, selective regulatory asymmetry resolves ancestral genomic conflicts by channeling stress-responsive pathways into lineage-specific functional trajectories. This mechanism establishes a link between genome duplication and ecological innovation.

## Materials and methods

### Sampling, sequencing, assembly, and annotation

**Fig. 1:**
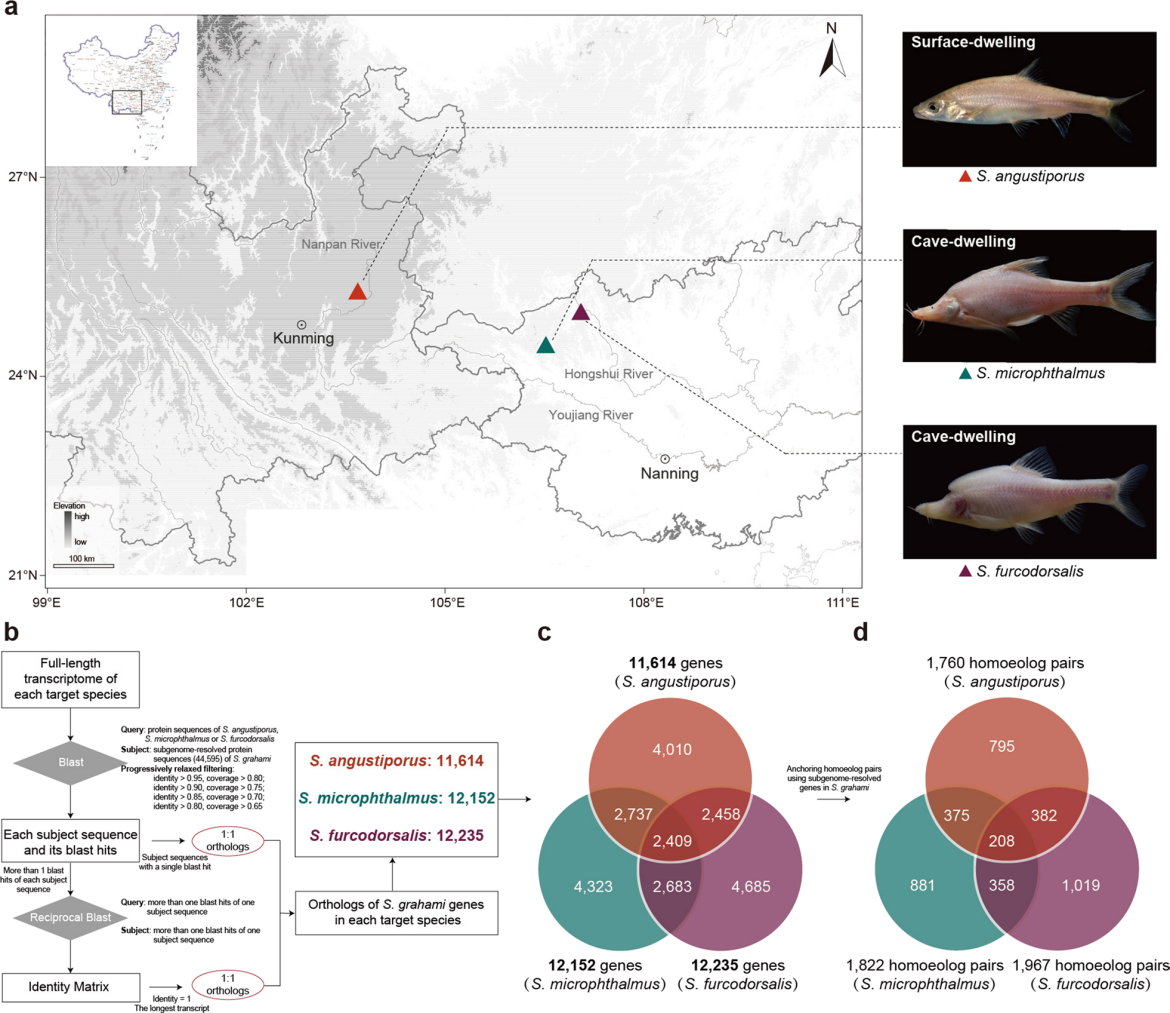
Sampling information and homoeolog pair identification based on full-length transcriptomes of three *Sinocyclocheilus* species. **a** Sampling information of three *Sinocyclocheilus* species in this study. **b** Identification pipeline of *S. grahami* orthologous genes in each species. **c** Venn diagram showing unique and shared orthologs (1:1:1) among the three species*.* **d** Venn diagram showing unique and shared homoeolog pairs (1:1:1) among the three species

Specimens of the three *Sinocyclocheilus* species were collected from Yunnan Province and Guangxi Zhuang Autonomous Region (Fig. 1a). For each species, three adult individuals were sampled. Brain, liver, muscle, and skin tissues were dissected and immediately frozen in liquid nitrogen for sequencing. The full-length transcriptomes of four mixed tissues were sequenced using the PacBio Sequel II platform. Additionally, brain-specific transcriptomes were sequenced using the DNBSEQ-T7RS platform (Nextomics Biosciences Co., Ltd., Wuhan, China). All procedures involving animals were approved by the Animal Care and Use Committee of the Institute of Zoology, Chinese Academy of Sciences (approval number: IOZ18002).

Raw subreads from the PacBio platform were processed using the Iso-Seq3 pipeline (https://github.com/PacificBiosciences/IsoSeq3) to assemble high-quality full-length transcriptomes. Circular consensus sequences (CCS) were generated using ccs v6.4.0 with default parameters. Full-length (FL) and non-full-length (NFL) reads were identified based on the presence or absence of 3′ and 5′ primers and a Poly(A) tail. Primer removal and poly(A) tail trimming were performed using Lima v2.9.0 and Refine v4.0.0, respectively, with default parameters. Finally, the full length non-chimeric reads (FLNC) sequences were clustered using cluster v4.0.0, followed by extraction of high-quality isoforms and redundancy removal using cd-hit v4.8.1 (identity > 99%). Transcriptome completeness after de-redundancy was assessed using BUSCO v5.3.0 [26].

Non-redundant transcript sequences (unigenes) were subjected to open reading frame (ORF) prediction using the ANGEL tool (https://github.com/PacificBiosciences/ANGEL) with default parameters. Coding (CDS) and corresponding amino acid sequences were extracted for functional analysis. Gene annotation was performed using BLAST v2.12 [27], with a significance cutoff of E-value < 1E−5 and other parameters set to default. Functional classification of unigenes was conducted using Gene Ontology (GO) [28] and Kyoto Encyclopedia of Genes and Genomes (KEGG) databases [29]. Transcription factors (TFs) were identified using AnimalTFDB v4.0 (https://guolab.wchscu.cn/AnimalTFDB4) and classified into TF gene families [30] using HMMER v3.0 [31]. Simple sequence repeats (SSRs) were detected using the MISA tool (http://pgrc.ipk-gatersleben.de/misa/), which categorizes microsatellite motifs into mono-, di-, tri-, tetra-, penta-, and hexa-nucleotide classes.

### Identification of orthologs and homoeolog pairs

To resolve subgenomic structure, the *S. grahami* genome [18] was partitioned into A and B subgenomes using *Cyprinus carpio* as a reference, based on BLASTN alignments [22]. BLAST v2.2.26 [27] was then used to align the predicted peptide sequences from the three *Sinocyclocheilus* species against the above subgenome-resolved protein sequences of *S. grahami* with an E-value threshold of 1E−3 (Fig. 1b). Hits were filtered using stringent criteria (“identity > 0.95 and coverage > 0.8”) to identify putative orthologs (Fig. 1b). Transcripts without BLAST hits were classified as 1:0 orthologs, while those with a single high-confidence hit were categorized as 1:1 orthologs (Fig. 1b). In cases where multiple hits were detected, the transcript showing the highest sequence identity and full-length alignment was retained as the best representative 1:1 ortholog. To increase ortholog recovery, the mapping results were further filtered using three progressively relaxed parameter sets: “identity > 0.9 and coverage > 0.75”; “identity > 0.85 and coverage > 0.7”; “identity > 0.8 and coverage > 0.65” (Fig. 1b). Orthologs identified under these relaxed thresholds were merged iteratively to expand the ortholog catalog between each species and the reference species (Fig. 1b). From these results, 1:1:1 orthologs shared among all three *Sinocyclocheilus* species were extracted (Fig. 1c). Homoeolog pairs specific to each species were subsequently identified from this ortholog set, providing a high-confidence set of 1:1 homoeologs for downstream subgenome expression analyses (Fig. 1d).

### Weighted gene co-expression network analysis (WGCNA)

High-quality transcriptome reads were generated by trimming raw sequencing data with fastp v0.12.4 [32] and alignment to species-specific CDS using BWA v0.7.17-r1188 [33]. Gene expression was quantified using Salmon v1.10.2 [34], with expression levels normalized to transcripts per million (TPM). Orthologous and homoeologous gene sets (Fig. 1c, d) were used to extract matched expression profiles across the three *Sinocyclocheilus* species. For each homoeologous gene pair, the copy exhibiting at least a twofold higher expression [20, 35] than the other was considered to have dominant expression. A two-sided chi-squared test, as described in previous studies [22, 35–37], was performed to test whether the dominant gene number in the A subgenome significantly differed from that in the B subgenome. WGCNA was performed using the R package WGCNA v1.72-1 [38], focusing on genes with non-zero TPM values in brain tissue. Co-expression networks were constructed independently for comparisons between *S. angustiporus* and *S. microphthalmus* and between *S. angustiporus* and *S. furcodorsalis*, to identify species-specific gene modules associated with cave adaptation. Modules exhibiting strong positive or negative correlations (correlation coefficients ≥ 0.70 or ≤  − 0.70) with statistically significant associations (*p* < 0.05) were retained for further analysis. Within each module, gene significance (GS) and module membership (MM) were calculated, and core module genes were defined by GS > 0.40 and MM > 0.50.

### Functional enrichment analysis

KEGG pathway enrichment analysis of differentially expressed genes (*p* < 0.05) was performed using the clusterProfiler v4.4.4 R package [39]. The top 10–15 enriched pathways were selected and visualized using the ggplot2 v3.5.1 R package [40].

## Results

### High-quality, full-length transcriptomes of three *Sinocyclocheilus* species

**Fig. 2:**
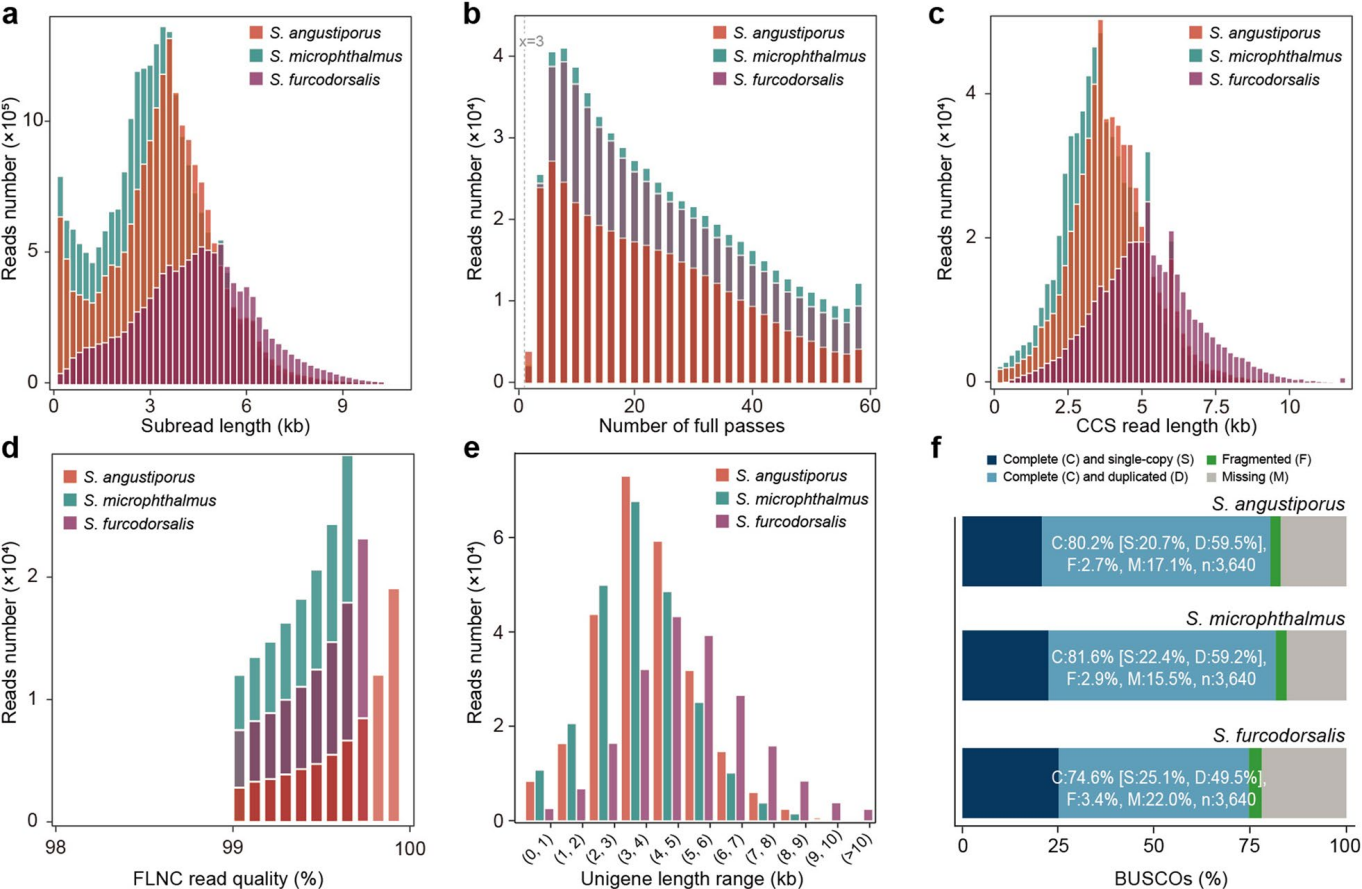
Quality evaluation for reads generated using the Iso-seq3 analysis pipeline. **a** Length distribution of subreads. **b** Length distribution of subreads with more than three full passes. **c** Length distribution of CCS reads. **d** Quality distribution of full length non-chimeric reads (FLNC) reads. **e** Length range distribution of unigenes. **f** BUSCO completeness metrics for full-length transcriptomes

High-fidelity PacBio Iso-Seq data were generated for the three *Sinocyclocheilus* species to construct high-resolution transcriptomic landscapes. Sequencing yielded 20,074,273, 23,654,573, and 11,589,089 subreads, with average lengths of 3.54, 3.23, and 4.63 kb and N50 lengths of 4.15, 3.87, and 5.26 kb, respectively (Fig. 2a and Table S1). Subsequent processing of subreads with more than three full passes produced 620,461, 683,050, and 401,523 CCS reads (Fig. 2b), with average lengths of 3.99, 3.71, and 5.21 kb and N50 lengths of 4.33, 4.12, and 5.64 kb, respectively (Fig. 2c and Table S1). FLNC transcripts were identified through poly(A) tail and primer detection, resulting in 613,077 (*S. angustiporus*), 676,574 (*S. microphthalmus*), and 398,596 (*S. furcodorsalis*) high-confidence transcripts (Table S1). After clustering and redundancy reduction, 256,272, 237,700, and 196,497 non-redundant unigenes were retained, each exhibiting sequence identity larger than 99% (Fig. 2d and Table S1). Length distribution analysis revealed that most unigenes exceeded 2 kb—90.41% in *S. angustiporus*, 86.89% in *S. microphthalmus*, and 95.32% in *S. furcodorsalis* (Fig. 2e and Table S3). BUSCO assessments confirmed transcriptome completeness, detecting 80.2% (single-copy: 20.7%, duplicated: 59.5%), 74.6% (single-copy: 22.4%, duplicated: 59.2%), and 81.6% (single-copy: 25.1%, duplicated: 49.5%) complete orthologs, respectively, suggesting high-quality full-length transcriptomes (Fig. 1f).

Coding sequence (CDS) prediction yielded 233,965 (*S. angustiporus*), 212,345 (*S. microphthalmus*), and 190,606 (*S. furcodorsalis*) CDSs in the three species, with length distributions of 90.97%–94.87% below 3.0 kb, 4.67–7.42% between 3.0 and 5.0 kb, and 0.46–1.61% exceeding 5.0 kb (Table S3). Notably, over 95% of CDSs encoded more than 1,200 amino acids across all species (Fig. S1 and Table S3). Analysis of TFs using AnimalTFDB v4.0 identified 57,548 TFs in *S. angustiporus*, 51,702 TFs in *S. furcodorsalis*, and 52,719 TFs in *S. microphthalmus*, distributed across 72 gene families (Fig. S2a and Table S4). In parallel, SSR profiling revealed a strong bias toward shorter repeat motifs, with mononucleotide and dinucleotide repeats accounting for over 60% and 25% of SSRs across all three species (Fig. S2b and Table S5).

### Convergent B-subgenome expression dominance in two cave-dwelling species

**Fig. 3:**
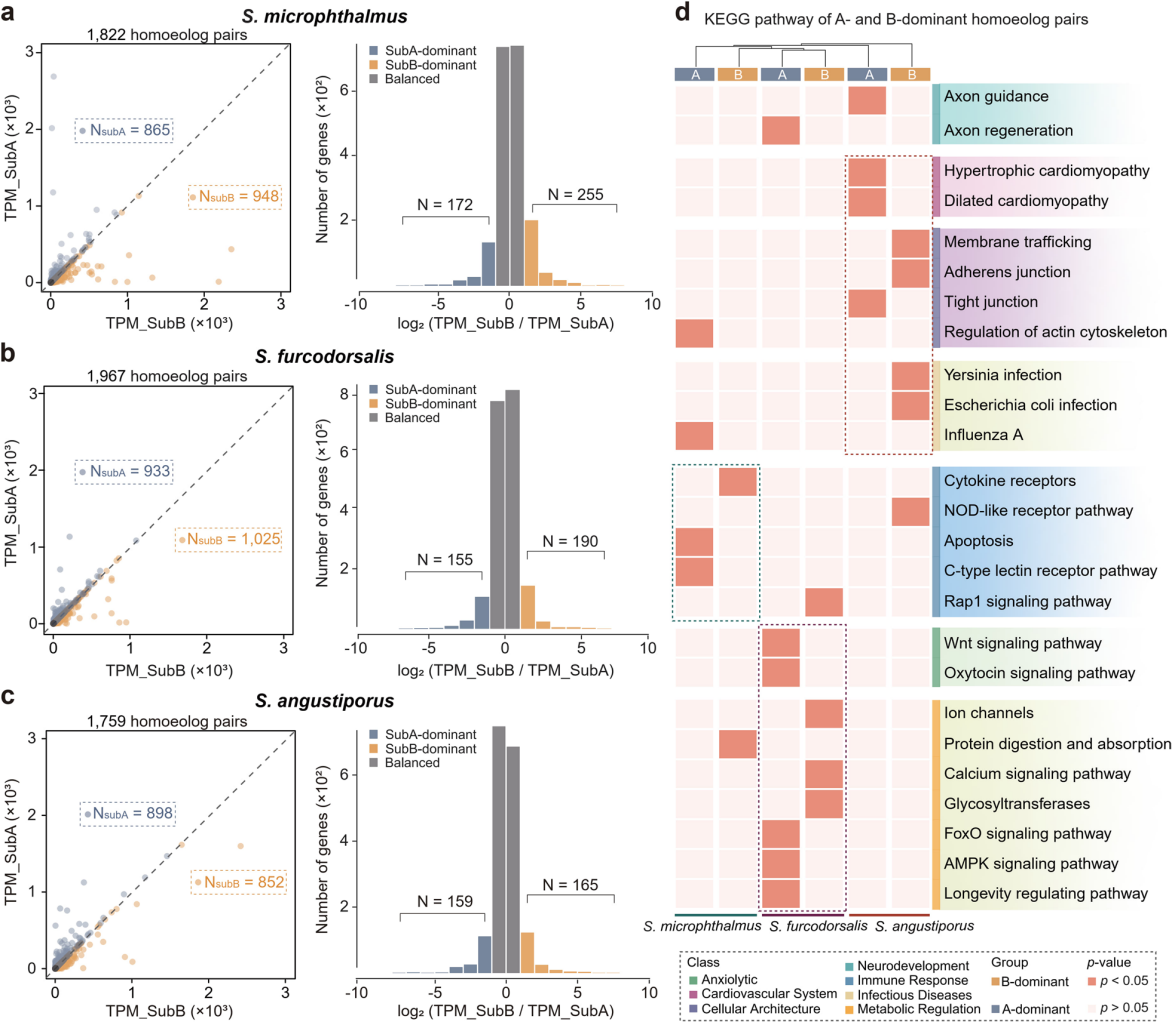
Expression divergence of homoeolog pairs across subgenomes in *S. angustiporus*, *S. microphthalmus*, and *S. furcodorsalis*. **a**–**c** Distribution of TPM_SubA/TPM_SubB ratios (left) and expression divergence of homoeologous genes (right) in brain tissue across three species. Dashed line (TPM_SubB/TPM_SubA = 1) indicates equal expression. Blue dots represent homoeolog pairs with a ratio > 1, orange dots denote those with reversed expression bias. Log_2_ (TPM_SubB/TPM_SubA) indicates degree of expression difference in homoeolog pairs. N values indicate number of homoeolog pairs with two-fold expression divergence. **d** KEGG enrichment analysis of SubA- and SubB-dominant homoeologs in two subgenomes across three species. Pathways are categorized into seven functional groups. Color gradient represents significance level of enrichment, with red indicating *p* < 0.05

To investigate subgenome regulatory asymmetry associated with cave adaptation, transcriptional profiling was conducted on 1,822, 1,967, and 1,759 high-confidence homoeolog pairs in *S. microphthalmus*, *S. furcodorsalis*, and *S. angustiporus*, respectively (Fig. 3a–c left). Comparative transcriptomic analyses revealed a consistent pattern of expression bias favoring the B subgenome in cave-adapted *Sinocyclocheilus* species, in contrast to the more balanced subgenome activity observed in their surface-dwelling counterpart. In *S. microphthalmus*, 948 homoeolog pairs showed elevated expression from the B subgenome relative to 865 from the A subgenome, while *S. furcodorsalis* exhibited a similar skew with 1,025 vs. 933 gene pairs, respectively (Fig. 3a–b, left). In comparison, *S. angustiporus* showed a slight shift toward A-subgenome expression (898 vs. 852 gene pairs), indicating no pronounced subgenomic bias (Fig. 3c, left). To delineate dominant expression patterns, homoeolog pairs with at least a two-fold expression bias toward one subgenome relative to its counterpart were classified as dominantly expressed, following established definitions [41, 42]. Notably, *S. microphthalmus* exhibited significant B-subgenome dominance, with 255 B-dominant versus 172 A-dominant homoeologs (χ^2^ = 16.1335, *p* = 5.9e-5) (Fig. 3a right and Table 1), while *S. furcodorsalis* showed a similar directional trend (190 vs. 155), although it did not reach statistical significance (χ^2^ = 3.5507, *p* = 0.0595) (Fig. 3b right and Table 1). In contrast, *S. angustiporus* displayed no significant subgenome dominance, with 165 B-dominant and 159 A-dominant homoeolog pairs (χ^2^ = 0.1111, *p* = 0.7389) (Fig. 3c right and Table 1). This convergent bias toward B-subgenome expression in both cave-adapted species, despite their phylogenetic divergence, suggests that subgenome dominance may represent a key regulatory mechanism. This mechanism may facilitate adaptation to the extreme conditions of subterranean environments. Notably, to rule out effects of the cutoff choice, we also tested a 1.5-fold cutoff. It identified more genes, but the functional enrichments matched those at twofold. We therefore used the commonly adopted twofold threshold (Fig. S3).

**Table 1:** Two-sided chi-squared test for dominant genes expressed in the brain tissue of each species

Species	Observed SubA	Observed SubB	Expected SubA	Expected SubB	χ^2^	df	*p-*value
*S. microphthalmus*	172.0	255.0	213.5	213.5	16.1335	1	5.9e−5
*S. furcodorsalis*	155.0	190.0	172.5	172.5	3.5507	1	0.0595
*S. angustiporus*	159.0	165.0	162.0	162.0	0.1111	1	0.7389

KEGG and GO functional enrichment analyses revealed striking contrasts in the biological roles of dominantly expressed homoeolog pairs across subgenomes between surface- and cave-dwelling species (Figs. 3d and S5). In the surface-dwelling *S. angustiporus*, dominant homoeolog pairs from both subgenomes were primarily enriched in cardiovascular function (e.g., hypertrophic cardiomyopathy and heart development), immune responses (e.g., *Yersinia* infection and defense response to virus), and molecular transport pathways (e.g., membrane trafficking), consistent with physiological demands imposed by open-water habitats and sustained mobility [43] (Figs. 3d and S5). In contrast, both cave-adapted species exhibited subgenome specialization aligned with subterranean ecological constraints. Dominant homoeolog pairs in the two species were significantly enriched in immune regulation, including lineage-specific activation of the T cell receptor signaling pathway and inflammatory response in *S. microphthalmus* and the Rap1 signaling pathway in *S. furcodorsalis*. Metabolic adaptations were also prominent, with overrepresented pathways such as protein digestion and absorption in *S. microphthalmus* and MAPK signaling in *S. furcodorsalis*, hallmarks of survival under nutrient-poor conditions [4, 44] (Fig. 3d). Comparative analyses further revealed species-specific divergence in the functional deployment of dominant homoeolog pairs. In *S. microphthalmus*, dominant homoeologs from the two subgenomes were primarily involved in immunological modulation. In *S. furcodorsalis*, however, dominant homoeologs were significantly enriched in anxiolytic and metabolic regulation, mainly via Wnt and MAPK signaling, pathways implicated in behavioral modulation and efficient energy utilization under metabolic constraint (Fig. 3d) [45]. These distinct subgenomic signatures reflect lineage-specific solutions to the physiological demands of cave life and underscore the role of subgenome partitioning in facilitating adaptive divergence.

Together, these results connect genome duplication to ecological adaptation in a testable way. Preferential use of the B subgenome seems to help these fishes cope with cave pressures (e.g., long-term food shortage, perpetual darkness, and shifts in pathogen exposure) by raising adaptive-immune activity and shifting metabolism toward more energy-efficient modes. In this way, the polyploid genome is not a fixed gene store but a modular system with built-in regulatory bias that can be selectively used during cave adaptation in *Sinocyclocheilus*.

### Distinct functional specialization of subgenome-dominant homoeologs

**Fig. 4:**
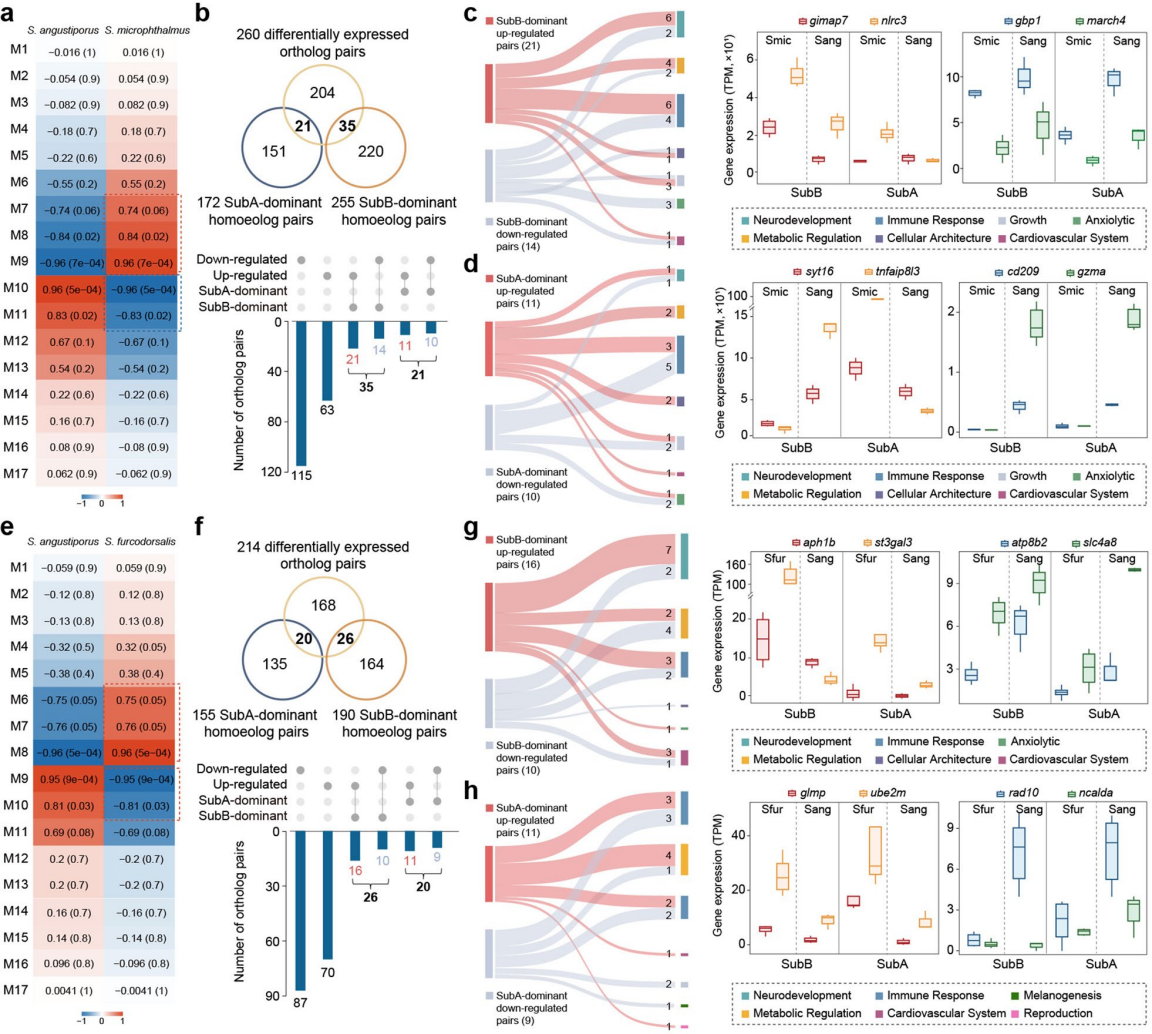
Differentially expressed homoeolog pairs exhibiting subgenome dominance in two cave-dwelling species. **a**, **e** Heatmaps showing modules from WGCNA comparing *S. angustiporus* with *S. microphthalmus* and *S. angustiporus* with *S. furcodorsalis*. Correlation coefficients |*r*|> 0.7 with *p* < 0.05 were considered significant. Red and blue dashed boxes indicate up- and down-regulated correlations, respectively. **b**, **f** Overlap of differentially expressed ortholog pairs (up- and down-regulated gene pairs) and subgenome-dominant homoeolog pairs (SubA and SubB) in *S. microphthalmus* and *S. furcodorsalis*, respectively. **c**, **d**, **g**, **h** Functional classification of SubA- and SubB-dominant homoeolog pairs (up-regulated in red and down-regulated in blue) and subgenome expression divergence of representative key genes in *S. microphthalmus* and *S. furcodorsalis*. Sang, *S. angustiporus*; Smic, *S. microphthalmus*; Sfur, *S. furcodorsalis*. SubA, subgenome A; SubB, subgenome B

To investigate the evolutionary implications of subgenome dominance in cave-adapted *Sinocyclocheilus* lineages, subgenome-resolved co-expression networks were constructed by comparing *S. angustiporus* with the cave-dwelling *S. microphthalmus* and *S. furcodorsalis* (Fig. 4a, e and S4a, e). In *S. microphthalmus*, three modules (M7–9) exhibited strong positive correlation with cave phenotype (*r* > 0.70, *p* ≤ 0.05), containing 900 up-regulated genes, while two modules (M10–11) were negatively associated (*r* <  − 0.70, *p* ≤ 0.05), encompassing 2,095 down-regulated genes. Gene filtering with MM (cor.MM > 0.50) and GS (cor.GS > 0.40) resolved 839 up-regulated and 1 757 down-regulated candidate genes (Figs. 4a and S4b, c). A parallel regulatory structure was observed in *S. furcodorsalis*, with three positively correlated (M6–8) and two negatively correlated modules (M9 and M10), corresponding to 703 up-regulated and 1,803 down-regulated genes (Figs. 4e and S4e, f). Cross-species comparison of these networks revealed conserved patterns of subgenome-biased expression among differentially expressed homoeolog pairs (Fig. 4b, f). Intersection with cross-species ortholog pairs (Fig. 1b–d) identified 260 and 214 differentially expressed gene pairs in *S. microphthalmus* and *S. furcodorsalis*, respectively (Fig. 4b, f). In *S. microphthalmus*, B-subgenome dominance (SubB-dominant) was evident in 35 gene pairs (21 up-regulated and 14 down-regulated), while A-subgenome dominance (SubA-dominant) was observed in 21 gene pairs (11 up-regulated and 10 down-regulated) (Fig. 4b). Similarly, *S. furcodorsalis* exhibited 26 SubB-dominant pairs (16 up-regulated and 10 down-regulated) and 20 SubA-dominant pairs (11 up-regulated and nine down-regulated) (Fig. 4f). The convergent transcriptional asymmetry toward B-subgenome activity across both cave-dwelling species highlights a recurrent regulatory signature potentially underpinning lineage-specific adaptation to subterranean environments.

To further elucidate the functional relevance of subgenome dominance in cave adaptation, ortholog pairs showing both lineage-specific differential expression and subgenome-biased expression in cave-dwelling species were subjected to functional classification (Fig. 4c, d, g, h, and Table S6). In *S. microphthalmus*, SubB-dominant gene pairs associated with cave-specific expression shifts were significantly enriched in immune-related pathways, particularly those linked to adaptive immune regulation. Among these, six up-regulated and four down-regulated orthologous gene pairs were identified, with notable involvement in immune effector functions (Fig. 4c). Notably, the two up-regulated pairs of *gimap7* (modulating apoptotic balance) [46] and *nlrc3* (T-cell response regulation) [47] were associated with adaptive cellular immune responses, while the two down-regulated pairs of *gbp1* (inflammasome activation) [48] and *march4* (antiviral innate immune signaling pathways) [49] were associated with innate immunity components (Fig. 4c). A-subgenome-dominant gene pairs in *S. microphthalmus* displayed a complementary immune profile. Up-regulated genes, such as *syt16* (B and T cell infiltration) [50] and *tnfaip8l3* (proliferation, inflammation, and cell death), indicated enhanced adaptive immunity [51], while down-regulated genes, including *cd209* (pathogen recognition receptor) [52] and *gzma* (cell death during phagocytosis), pointed to attenuated innate immune responsiveness [53] (Fig. 4d). These patterns reveal a subgenome-dependent reorganization of immune investment strategies, with differential emphasis on adaptive versus innate immunity components. The results suggest that subgenome-biased expression is intricately linked to functional specialization in cave environments, where altered pathogen exposure and metabolic constraints may have reshaped immune priorities.

In *S. furcodorsalis*, subgenome-biased expression revealed functional specialization toward neurodevelopmental delay and metabolic adaptation, consistent with selective pressures in nutrient-deprived, lightless cave environments. Analysis of B-subgenome-dominant orthologs identified seven up-regulated gene pairs enriched in neurodevelopmental pathways. Notably, *aph1b* (neurodevelopmental disorders) [54] and *st3gal3* (intellectual disability and behavioral disorders) [55] were implicated in disrupted synaptic maturation. This transcriptional signature corresponds with established observations of reduced visual system development in cave-dwelling fish species [56]. In parallel, four down-regulated B-dominant orthologs, including *atp8b2* (ATP-dependent phospholipid/metal ion transporter) [57] and *slc9a7* (Na + /H + homeostasis maintenance) [58], were linked to metabolic regulation. This pattern may potentially reflect physiological adjustments to the chronic energy scarcity typical of subterranean habitats [59] (Fig. 4g). Functional profiling of A-subgenome-dominant orthologs revealed complementary patterns. Four up-regulated orthologs were primarily associated with lipid metabolism, including *glmp* (fatty acid uptake and lipogenesis) [60] and *ube2m* (lipid accumulation and obesity phenotypes) [61]. In contrast, three down-regulated A-dominant orthologs were functionally linked to neural development and intracellular transport, such as *rad10* (vesicle trafficking mediator) [62] and *ncalda* (regulator of neurogenesis) [63], reinforcing the transcriptomic signature of attenuated neurodevelopment observed in B-subgenome expression (Fig. 4h). Comparative analysis with surface-dwelling species further revealed divergent subgenome-biased expression in cavefish lineages, shaped by cave-specific environmental pressures (e.g., light deprivation and nutrient scarcity), ultimately driving species-specific adaptations in immune regulation and metabolic reprogramming.

Together, these results strengthen the link between genome duplication and ecological adaptation. The key signals concentrate in WGD-derived homoeologs, indicating that duplication provides the regulatory space for functional reassignment under cave constraints. Moreover, the repeatable B-subgenome bias in two independently cave-adapted lineages points to a shared regulatory solution. Functionally, duplicated copies are differentially deployed: B-linked homoeologs elevate adaptive-immune and stress-response programs, while A-linked sets redistribute metabolism toward more energy-efficient modes under long-term food shortage and shifting pathogen exposure. Thus, WGD supplies the substrate for subgenome-level partitioning that translates molecular bias into cave-relevant physiological adaptation.

## Discussion

Comparative subgenome-resolved transcriptomic analysis revealed convergent B-subgenome dominance and lineage-specific functional divergence in cave-adapted *Sinocyclocheilus* species. This pronounced regulatory asymmetry underscores subgenome dominance as a key mechanism facilitating adaptation to the extreme conditions of subterranean ecosystems. It also provides novel insights into how polyploid genomic architectures mediate evolutionary innovation under environmental stress.

The consistent expression bias toward the B subgenome across independently evolved cave lineages mirrors trends in polyploid relatives such as common carp [20, 35] and goldfish [22]. Common carp and goldfish share the Cyprinidae-specific whole-genome duplication (Cs4R) event with *Sinocyclocheilus* [20, 22], and their A/B subgenomes are macro-syntenically orthologous across Cyprinidae. In these species, expression is biased toward the B subgenome, with B-dominant homoeologs enriched for hypoxia/stress response, oxidoreductase activity, hydrolase activity, and DNA repair [20, 22]. A similar pattern of subgenome dominance is also observed in cave *Sinocyclocheilus* species at the chromosome level, where the D subgenome (B subgenome in this study) exhibits gene-retention and expression dominance corresponding to adaptive functions [64]. Consistent with this framework, population-genomic analyses in cave-dwelling *S. microphthalmus* also show significantly higher SNP density in the B(H) subgenome at the level of syntenic blocks and homoeologs [65]. This pattern is consistent with our observation of recurrent B-subgenome bias in cave *Sinocyclocheilus* and supports the view that the B subgenome serves as a preferential regulatory reservoir recruited under cave-specific selection. However, it contrasts with the balanced subgenome activity in surface-dwelling *S. angustiporus*. This pattern suggests that the B subgenome may be a preferential regulatory reservoir recruited under cave-specific selection. The asymmetric expression likely reflects ancestral genomic incompatibilities from the allopolyploid origin of *Sinocyclocheilus* [20, 21], in which retention of stress-responsive loci is biased toward one parental subgenome. Outside vertebrates, allopolyploid plants adapted to harsh environments show similar subgenome-level functional specialization. Similarly, enrichment of immune and metabolic regulators in B-subgenome-dominant genes resembles findings in polyploid extremophiles such as *Trifolium repens* [66] and *Sesbania cannabina* [67]. In these species, subgenome-specific expression of stress response loci (e.g., *RafS* and *AT1*) supports tolerance to cold and saline-alkaline soils. Likewise, the xerophytic allopolyploid *Lespedeza potaninii* shows consistent B-subgenome dominance across multiple tissues, with functional enrichment in carbohydrate and phenylpropanoid metabolism linked to survival under arid, nutrient-limited conditions [68]. These cross-taxonomic parallels suggest that subgenome-biased regulation can channel adaptive shifts in energy allocation, stress response, and tissue remodeling, key processes also implicated in cave adaptation.

The two subgenomes show lineage-specific functional specialization: adaptive immune regulation in *S. microphthalmus* and metabolic reprogramming in *S. furcodorsalis*. This highlights the versatility of polyploid genomes in addressing distinct ecological challenges. In *S. microphthalmus*, immune-regulatory genes from both subgenomes are elevated, including *gimap7*, *nlrc3*, *syt16*, and *tnfaip8l3* (Fig. 4c–d). This pattern suggests a prioritization of adaptive immunity over metabolically expensive innate defenses. At the sequence level, the patterns parallel the expression shift. In *S. microphthalmus*, the immune gene *fcgbp* shows a lower density of nonsynonymous SNPs in the H (B) copy, whereas the nervous-system genes *cacna2d2*, *pcdha3*, and *pcdhac2* exhibit higher nonsynonymous SNP densities in the L (A) copy [65]. The dominant copy tends to be more highly expressed and under stronger purifying selection (fewer nonsynonymous changes), these patterns imply B-subgenome expression dominance for *fcgbp* and A-subgenome dominance for *cacna2d2*, *pcdha3*, and *pcdhac2*. This shift likely reflects trade-offs imposed by open aquatic habitats, where pathogen exposure is higher, requiring efficient immune surveillance that minimizes energetic cost. The semi-open cave habitats of *S. microphthalmus* are connected to underground rivers linked with surface waters [69, 70]. These habitats may have relatively high pathogen diversity compared to fully enclosed caves, leading to increased pathogen exposure. Innate immunity, however, is energetically costly and can lead to chronic inflammation [71–73], whereas adaptive immunity offers a more efficient long-term solution by relying on memory cells to recognize and respond to pathogens more effectively with less energy consumption [74, 75]. This is particularly advantageous in nutrient-poor cave environment for cavefish. Similar immune remodelling has been reported in other cavefish, such as *A. mexicanus* and *Triplophysa rosa*, where T-cell-based immune strategies dominate [76, 77]. The observed immune allocation aligns with the immunocompetence handicap hypothesis [78], wherein immune function is selectively modulated to balance survival and energy efficiency under resource limitations. In contrast, *S. furcodorsalis* exhibits metabolic specialization optimized for trophically constrained environments. Down-regulation of ion-transport regulators such as *atp8b2* and *slc9a7* (Fig. 4f) suggests suppression of energetically costly ATP-dependent processes, a strategy observed in hibernating mammals during extended fasting [79]. Concurrent up-regulation of lipid metabolic genes from the A subgenome, including *glmp* and *ube2m* (Fig. 4g–h), supports enhanced lipid storage capacity. This metabolic economy is further reinforced by the down-regulation of neurodevelopmental genes such as *aph1b* and *st3gal3*, consistent with reduced sensory investment, a well-established feature of cave adaptation [56, 80]. These traits parallel phenotypic trends in *A. mexicanus*, including reduced sociality [81], aggressiveness [82], and sleep [83], which collectively reduce energy expenditure under persistent resource scarcity. Consistent with these molecular patterns, *S. furcodorsalis* has no eyes and has far fewer neuromasts, reflecting adaptation to the perpetual darkness in caves [84]. In addition, it also has a dorsal humpback that lacks bone and is mainly composed of adipose tissue [85]. These traits suggest an adaptive energy reallocation strategy, whereby resources are moved away from vision and sensory systems toward long-term fat storage, helping the fish survive in caves where food is persistently scarce. At the genome scale, these functional shifts align with subgenome-level regulatory asymmetry observed in cave lineages [64], suggesting that adaptive immune and metabolic remodeling can be controlled through the dominant subgenome.

Collectively, these findings establish *Sinocyclocheilus* as a model system for understanding how polyploid genomes resolve evolutionary trade-offs during extreme environmental constraints. The consistent dominance of subgenome B in cave-dwelling species highlights the role of ancestral polyploid subgenomes as reservoirs of adaptive capacity, selectively deployed to meet ecological challenges. These findings contribute to the growing intersection of polyploid genomics and extremophile research, offering a framework for investigating the genetic basis of resilience in rapidly shifting environments. Nonetheless, key questions remain. First, the role of post-polyploidization diploidization, such as fractionation bias and chromosomal rearrangements, in shaping subgenome architecture remains unclear. Second, broader comparative studies with additional cave-adapted polyploid species and chromosome-level assemblies are needed to separate lineage-specific adaptations from general patterns.

## Supplementary Information

Additional file 1.

## Data Availability

The raw full-length transcriptome data (GSA: CRA023322) generated in this study have been deposited in the Genome Sequence Archive (GSA) in the National Genomics Data Center, China National Center for Bioinformation/Beijing Institute of Genomics, Chinese Academy of Sciences, and are publicly accessible at https://ngdc.cncb.ac.cn/gsa. All data used in the study are provided in Tables S1 and S2.

## References

[1] Van de Peer Y, Ashman T-L, Soltis PS, Soltis DE. Polyploidy: an evolutionary and ecological force in stressful times. Plant Cell. 2021;33:11–26. 33751096 10.1093/plcell/koaa015 PMC8136868

[2] Van de Peer Y, Mizrachi E, Marchal K. The evolutionary significance of polyploidy. Nat Rev Genet. 2017;18:411–24. 28502977 10.1038/nrg.2017.26

[3] Li X, Wang M, Zou M, Guan X, Xu S, Chen W, et al. Recent and recurrent autopolyploidization fueled diversification of snow carp on the Tibetan Plateau. Mol Biol Evol. 2024;41:msae221. 39437268 10.1093/molbev/msae221 PMC11542630

[4] Swaminathan A, Xia F, Rohner N. From darkness to discovery: evolutionary, adaptive, and translational genetic insights from cavefish. Trends Genet. 2024;40:24–38. 38707509 10.1016/j.tig.2023.10.002 PMC11068324

[5] Larson DJ, Middle L, Vu H, Zhang W, Serianni AS, Duman J, et al. Wood frog adaptations to overwintering in Alaska: new limits to freezing tolerance. J Exp Biol. 2014;217:2193–200. 24737762 10.1242/jeb.101931

[6] Bright M, Lallier FH. The biology of vestimentiferan tubeworms. Oceanogr Mar Biol. 2010;48:213–66.

[7] Tappe DT. Natural history of the Tulare Kangaroo Rat. J Mammal. 1941;22:117–48.

[8] Jiang W-S, Li J, Xiang H-M, Sun C, Chang J-B, Yang J-X. Comparative analysis and phylogenetic and evolutionary implications of mitogenomes of Chinese *Sinocyclocheilus* cavefish (Cypriniformes: Cyprinidae). Zool Res. 2023;44:779–81. 37464935 10.24272/j.issn.2095-8137.2022.439 PMC10415761

[9] Xiao M-Y, Wang J-J, Luo T, Zhou J-J, Xiao N, Zhou J. *Sinocyclocheilus xingrenensis* (Cypriniformes, Cyprinidae), a new underground fish from Guizhou Province, Southeastern China. Zoosyst Evol. 2025;101:419–36.

[10] Luo Q, Tang Q, Deng L, Duan Q, Zhang R. A new cavefish of *Sinocyclocheilus* (Teleostei: Cypriniformes: Cyprinidae) from the Nanpanjiang River in Guizhou, China. J Fish Biol. 2024;104:484–96. 37344383 10.1111/jfb.15490

[11] Shao W-H, Cheng G-Y, Lu X-L, Zhou J-J, Zeng Z-X. Description of a new troglobitic *Sinocyclocheilus* (Pisces, Cyprinidae) species from the upper Yangtze River Basin in Guizhou, South China. Zoosyst Evol. 2024;100:515–29.

[12] Fan C, Wang M, Wang J-J, Luo T, Zhou J-J, Xiao N, et al. *Sinocyclocheilus xiejiahuai* (Cypriniformes, Cyprinidae), a new cave fish with extremely small population size from western Guizhou, China. ZooKeys. 2024;1214:119. 39397882 10.3897/zookeys.1214.127629 PMC11467493

[13] Xu C, Luo T, Zhou J-J, Wu L, Zhao X-R, Yang H-F, et al. *Sinocyclocheilus longicornus* (Cypriniformes, Cyprinidae), a new species of microphthalmic hypogean fish from Guizhou, Southwest China. ZooKeys. 2023;1141:1. 37234961 10.3897/zookeys.1141.91501 PMC10208810

[14] Zhao Y, Zhang C Endemic fishes of *Sinocyclocheilus* (Cypriniformes: Cyprinidae) in China—Species diversity, cave adaptation, systematics and zoogeography. Science Press, Beijing. 2009.

[15] Zhao YH, Gozlan RE, Zhang CG. Out of sight out of mind: current knowledge of Chinese cave fishes. J Fish Biol. 2011;79:1545–62. 22136239 10.1111/j.1095-8649.2011.03066.x

[16] Borowsky R. Cavefishes. Curr Biol. 2018;28:R60–4. 29374443 10.1016/j.cub.2017.12.011

[17] Trajano E, de Carvalho MR. Towards a biologically meaningful classification of subterranean organisms: a critical analysis of the Schiner-Racovitza system from a historical perspective, difficulties of its application and implications for conservation. Subterr Biol. 2017;22:1–26.

[18] Yang J, Chen X, Bai J, Fang D, Qiu Y, Jiang W, et al. The *Sinocyclocheilus* cavefish genome provides insights into cave adaptation. BMC Biol. 2016. 10.1186/s12915-015-0223-4. 10.1186/s12915-015-0223-4 PMC4698820 26728391

[19] Jeffery WR *Astyanax mexicanus*: a vertebrate model for evolution, adaptation, and development in caves. In Encyclopedia of Caves*.* 2019. pp 85–93.

[20] Xu P, Xu J, Liu G, Chen L, Zhou Z, Peng W, et al. The allotetraploid origin and asymmetrical genome evolution of the common carp *Cyprinus carpio*. Nat Commun. 2019;10:4625. 31604932 10.1038/s41467-019-12644-1 PMC6789147

[21] Li X, Guo B. Substantially adaptive potential in polyploid cyprinid fishes: evidence from biogeographic, phylogenetic and genomic studies. Proc R Soc B Biol Sci. 2020. 10.1098/rspb.2019.3008. 10.1098/rspb.2019.3008 PMC7031659 32075533

[22] Li J-T, Wang Q, Huang Yang M-D, Li Q-S, Cui M-S, Dong Z-J, et al. Parallel subgenome structure and divergent expression evolution of allo-tetraploid common carp and goldfish. Nat Genet. 2021;53:1493–503. 34594040 10.1038/s41588-021-00933-9 PMC8492472

[23] Ebadi M, Bafort Q, Mizrachi E, Audenaert P, Simoens P, Van Montagu M, et al. The duplication of genomes and genetic networks and its potential for evolutionary adaptation and survival during environmental turmoil. Proc Natl Acad Sci USA. 2023;120:e2307289120. 37788315 10.1073/pnas.2307289120 PMC10576144

[24] Chen SY, Zhang RD, Feng JG, Xiao H, Li WX, Zan RG, et al. Exploring factors shaping population genetic structure of the freshwater fish *Sinocyclocheilus grahami* (Teleostei, Cyprinidae). J Fish Biol. 2009;74:1774–86. 20735670 10.1111/j.1095-8649.2009.02204.x

[25] Xiao H, Chen S-y, Liu Z-m, Zhang R-d, Li W-x, Zan R-g, et al. Molecular phylogeny of *Sinocyclocheilus* (Cypriniformes: Cyprinidae) inferred from mitochondrial DNA sequences. Mol Phylogenet Evol. 2005;36:67–77. 15904857 10.1016/j.ympev.2004.12.007

[26] Manni M, Berkeley MR, Seppey M, Simão FA, Zdobnov EM. BUSCO update: novel and streamlined workflows along with broader and deeper phylogenetic coverage for scoring of eukaryotic, prokaryotic, and viral genomes. Mol Biol Evol. 2021;38:4647–54. 34320186 10.1093/molbev/msab199 PMC8476166

[27] Boratyn GM, Schäffer AA, Agarwala R, Altschul SF, Lipman DJ, Madden TL. Domain enhanced lookup time accelerated BLAST. Biol Direct. 2012;7:1–14. 22510480 10.1186/1745-6150-7-12 PMC3438057

[28] Ashburner M, Ball CA, Blake JA, Botstein D, Butler H, Cherry JM, et al. Gene ontology: tool for the unification of biology. Gene Ontol Consort Nat Genet. 2000;25:25–9. 10.1038/75556 PMC3037419 10802651

[29] Kanehisa M, Goto S, Kawashima S, Okuno Y, Hattori M. The KEGG resource for deciphering the genome. Nucleic Acids Res. 2004;32:D277–80. 14681412 10.1093/nar/gkh063 PMC308797

[30] Shen WK, Chen SY, Gan ZQ, Zhang YZ, Yue T, Chen MM, et al. AnimalTFDB 4.0: a comprehensive animal transcription factor database updated with variation and expression annotations. Nucleic Acids Res. 2023;51:D39–45. 36268869 10.1093/nar/gkac907 PMC9825474

[31] Eddy SR. Profile hidden Markov models. Bioinformatics. 1998;14:755–63. 9918945 10.1093/bioinformatics/14.9.755

[32] Chen S, Zhou Y, Chen Y, Gu J. fastp: an ultra-fast all-in-one FASTQ preprocessor. Bioinformatics. 2018;34:i884–90. 30423086 10.1093/bioinformatics/bty560 PMC6129281

[33] Li H Aligning sequence reads, clone sequences and assembly contigs with BWA-MEM. arXiv. 2013.

[34] Patro R, Duggal G, Love MI, Irizarry RA, Kingsford C. Salmon provides fast and bias-aware quantification of transcript expression. Nat Methods. 2017;14:417–9. 28263959 10.1038/nmeth.4197 PMC5600148

[35] Wang M, Li X, Wang C, Zou M, Yang J, Li XD, et al. Asymmetric and parallel subgenome selection co-shape common carp domestication. BMC Biolog. 2024;22:4. 10.1186/s12915-023-01806-9 PMC10762839 38166816

[36] Fang C, Jiang N, Teresi SJ, Platts AE, Agarwal G, Niederhuth C, et al. Dynamics of accessible chromatin regions and subgenome dominance in octoploid strawberry. Nat Commun. 2024;15:2491. 38509076 10.1038/s41467-024-46861-0 PMC10954716

[37] Huang X, Wang Y, Zhang S, Pei L, You J, Long Y, et al. Epigenomic and 3D genomic mapping reveals developmental dynamics and subgenomic asymmetry of transcriptional regulatory architecture in allotetraploid cotton. Nat Commun. 2024;15:10721. 39730363 10.1038/s41467-024-55309-4 PMC11680999

[38] Langfelder P, Horvath S. WGCNA: an R package for weighted correlation network analysis. BMC Bioinform. 2008;9:1–13. 10.1186/1471-2105-9-559 PMC2631488 19114008

[39] Wu T, Hu E, Xu S, Chen M, Guo P, Dai Z, et al. clusterProfiler 4.0: a universal enrichment tool for interpreting omics data. The Innovation. 2021. 10.1016/j.xinn.2021.100141. 10.1016/j.xinn.2021.100141 PMC8454663 34557778

[40] Payne R. Wiley interdisciplinary reviews: computational statistics. GenStat. 2009;1:255–8. 10.1002/wics.45 PMC2898213 20625470

[41] Schnable JC, Springer NM, Freeling M. Differentiation of the maize subgenomes by genome dominance and both ancient and ongoing gene loss. Proc Natl Acad Sci USA. 2011;108:4069–74. 21368132 10.1073/pnas.1101368108 PMC3053962

[42] Woodhouse MR, Cheng F, Pires JC, Lisch D, Freeling M, Wang X. Origin, inheritance, and gene regulatory consequences of genome dominance in polyploids. Proc Natl Acad Sci USA. 2014;111:5283–8. 24706847 10.1073/pnas.1402475111 PMC3986174

[43] Chen H-Y, Li C-Q, Chen S-Y, Xiao H. Metagenomic analysis reveals hidden links between gut microbes and habitat adaptation among cave and surface dwelling *Sinocyclocheilus* species. Zool Res. 2023;44:793. 37464937 10.24272/j.issn.2095-8137.2022.195 PMC10415777

[44] Bowden TJ. Modulation of the immune system of fish by their environment. Fish Shellfish Immunol. 2008;25:373–83. 18562213 10.1016/j.fsi.2008.03.017

[45] Yang J, Ueharu H, Mishina Y. Energy metabolism: a newly emerging target of BMP signaling in bone homeostasis. Bone. 2020;138:115467. 32512164 10.1016/j.bone.2020.115467 PMC7423769

[46] Sivanandam AB. Structural and biochemical analysis of GTPases of immunity-associated proteins (GIMAPs) and their interaction partners. Germany: Freie Universität Berlin; 2015.

[47] Chang MX, Xiong F, Wu XM, Hu YW. The expanding and function of *NLRC3* or *NLRC3*-like in teleost fish: recent advances and novel insights. Dev Comp Immunol. 2021;114:103859. 32896535 10.1016/j.dci.2020.103859

[48] Shi T, Huang L, Zhou Y, Tian J. Role of *GBP1* in innate immunity and potential as a tuberculosis biomarker. Sci Rep. 2022;12:11097. 35773466 10.1038/s41598-022-15482-2 PMC9247026

[49] Zheng C. The emerging roles of the MARCH ligases in antiviral innate immunity. Int J Biol Macromol. 2021;171:423–7. 33428955 10.1016/j.ijbiomac.2020.12.207

[50] Chen J, Wang Z, Wang W, Ren S, Xue J, Zhong L, et al. *SYT16* is a prognostic biomarker and correlated with immune infiltrates in glioma: a study based on TCGA data. Int Immunopharmacol. 2020;84:106490. 32289666 10.1016/j.intimp.2020.106490

[51] Lou Y, Liu S. The *TIPE* (*TNFAIP8*) family in inflammation, immunity, and cancer. Mol Immunol. 2011;49:4–7. 21924498 10.1016/j.molimm.2011.08.006

[52] Barreiro LB, Patin E, Neyrolles O, Cann HM, Gicquel B, Quintana-Murci L. The heritage of pathogen pressures and ancient demography in the human innate-immunity CD209/CD209L region. Am J Hum Genet. 2005;77:869–86. 16252244 10.1086/497613 PMC1271393

[53] Hochegger K, Eller P, Rosenkranz AR. Granzyme A: an additional weapon of human polymorphonuclear neutrophils (PMNs) in innate immunity? Blood. 2004;103:1176–1176. 14729659 10.1182/blood-2003-10-3708

[54] Park YH, Pyun J-M, Hodges A, Jang J-W, Bice PJ, Kim S, et al. Dysregulated expression levels of *APH1B* in peripheral blood are associated with brain atrophy and amyloid-β deposition in Alzheimer’s disease. Alzheimers Res Ther. 2021;13:1–10. 34732252 10.1186/s13195-021-00919-z PMC8567578

[55] Khamirani HJ, Zoghi S, Faghihi F, Dastgheib SA, Hassanipour H, Tabei SMB, et al. Phenotype of *ST3GAL3* deficient patients: a case and review of the literature. Eur J Med Genet. 2021;64:104250. 34022416 10.1016/j.ejmg.2021.104250

[56] Meredith R. Sensitive and critical periods during neurotypical and aberrant neurodevelopment: a framework for neurodevelopmental disorders. Neurosci Biobehav Rev. 2015;50:180–8. 25496903 10.1016/j.neubiorev.2014.12.001

[57] Wei L, Ji L, Han S, Xu M, Yang X Construction and validation of a prognostic model of metabolism-related genes driven by somatic mutation in bladder cancer. Front Biosci. 2023;28(10):242. (Landmark edition) 10.31083/j.fbl2810242 37919060

[58] Pedersen S, Counillon L. The SLC9A-C mammalian Na+/H+ exchanger family: molecules, mechanisms, and physiology. Physiol Rev. 2019. 10.1152/physrev.00028.2018. 10.1152/physrev.00028.2018 31507243

[59] Ortiz M, Legatzki A, Neilson JW, Fryslie B, Nelson WM, Wing RA, et al. Making a living while starving in the dark: metagenomic insights into the energy dynamics of a carbonate cave. ISME J. 2014;8:478–91. 24030597 10.1038/ismej.2013.159 PMC3906820

[60] Kong XY, Kase ET, Herskedal A, Schjalm C, Damme M, Nesset CK, et al. Lack of the lysosomal membrane protein, GLMP, in mice results in metabolic dysregulation in liver. PLoS ONE. 2015;10:e0129402. 26047317 10.1371/journal.pone.0129402 PMC4457871

[61] Lu X, Kong X, Wu H, Hao J, Li S, Gu Z, et al. UBE2M-mediated neddylation of *TRIM21* regulates obesity-induced inflammation and metabolic disorders. Cell Metab. 2023;35:1390-1405. e1398. 37343564 10.1016/j.cmet.2023.05.011

[62] Zou W, Yadav S, DeVault L, Jan YN, Sherwood DR. RAB-10-dependent membrane transport is required for dendrite arborization. PLoS Genet. 2015;11:e1005484. 26394140 10.1371/journal.pgen.1005484 PMC4578882

[63] Upadhyay A, Hosseinibarkooie S, Schneider S, Kaczmarek A, Torres-Benito L, Mendoza-Ferreira N, et al. Neurocalcin delta knockout impairs adult neurogenesis whereas half reduction is not pathological. Front Mol Neurosci. 2019;12:19. 30853885 10.3389/fnmol.2019.00019 PMC6396726

[64] Mao T, Liu Y, Svardal H, Vasconcellos M, Yang J, Yang L, He S, Meegaskumbura M Chromosomal restructuring and subgenome divergence drive post-polyploid adaptive diversification in *Sinocyclocheilus* cavefish. bioRxiv 2025:2025.2009. 2022.677718.

[65] Montero‐Mendieta S, Wang Y, Wang C, Meng F, Zhao Y, Li X, et al. Doubled genomes, divergent fates: genomic insights into diversification in an allotetraploid cavefish. Mol Ecol. 2025. 10.1111/mec.70118. 10.1111/mec.70118 40999710

[66] Fechete LI, Larking AC, Heslop A, Hannaford R, Anderson CB, Hong W, et al. Harnessing cold adaptation for postglacial colonisation: galactinol synthase expression and raffinose accumulation in a polyploid and its progenitors. Plant Cell Environ. 2024;47:4014–30. 38873953 10.1111/pce.15009

[67] Luo H, Wang X, You C, Wu X, Pan D, Lv Z, et al. Telomere-to-telomere genome of the allotetraploid legume Sesbania cannabina reveals transposon-driven subgenome divergence and mechanisms of alkaline stress tolerance. Sci China Life Sci. 2024;67:149–60. 37897613 10.1007/s11427-023-2463-y

[68] Qi Y, Pan X, Yunyue X, Lijun C, Fan W, Shengsheng W, et al. The genome of *Lespedeza potaninii* reveals biased subgenome evolution and drought adaptation. Plant Physiol. 2024;195(4):2829–42. 38758114 10.1093/plphys/kiae283

[69] Zhao Y, Zhao Y. ZHAO Y: The population diversity and conservation of endangered chinese cavefish Sinocyclocheilus microphthalmus. Baoding: Hebei University; 2021.

[70] Li W, Wu D, Chen A, Tao J. The preliminary investigation of geographical distribution and ecological adaptation for cave environment of *Sinocyclocheilus rhinocerous*—a cyprinid fish. Yunnan nong ye da xue xue bao = J Yunnan Agric Univ. 2000;15:1–4.

[71] Jeljeli MM, Adamopoulos IE. Innate immune memory in inflammatory arthritis. Nat Rev Rheumatol. 2023;19:627–39. 37674048 10.1038/s41584-023-01009-0 PMC10721491

[72] Tercan H, Riksen NP, Joosten LAB, Netea MG, Bekkering S. Trained Immunity : long-term adaptation in innate immune responses. Arteriosclerosis. 2020;41(1):55–61. 10.1161/ATVBAHA.120.314212 33086868

[73] Hajishengallis G, Li X, Mitroulis I, Chavakis T. Trained innate immunity and its implications for mucosal immunity and inflammation. Adv Exp Med Biol. 2019;1197:192. 10.1007/978-3-030-28524-1_2 PMC6986364 31732931

[74] Mcdaniel MM, Meibers HE, Pasare C. Innate control of adaptive immunity and adaptive instruction of innate immunity: bi-directional flow of information. Curr Opin Immunol. 2021;73:25–33. 34425435 10.1016/j.coi.2021.07.013 PMC8648974

[75] Vincenzo B, Asif IJ, Nikolaos P, Francesco M. Adaptive immunity and inflammation. Int J Inflam. 2015;2015:575406. 25793142 10.1155/2015/575406 PMC4352488

[76] Ni L, Jin L, Zeng M, Xu Y, Wang Y, Peng Z. The cavefish *Triplophysa rosa* has a well-developed adaptive immune system: evidence from histological and comparative genomic analysis. Aquaculture. 2024;581:740395.

[77] Peuß R, Box AC, Chen S, Wang Y, Tsuchiya D, Persons JL, et al. Adaptation to low parasite abundance affects immune investment and immunopathological responses of cavefish. Nat Ecol Evol. 2020;4:1416–30. 32690906 10.1038/s41559-020-1234-2 PMC11062081

[78] Sheldon BC, Verhulst S. Ecological immunology: costly parasite defences and trade-offs in evolutionary ecology. Trends Ecol Evol. 1996;11:317–21. 21237861 10.1016/0169-5347(96)10039-2

[79] Storey KB, Storey JM. Metabolic rate depression in animals: transcriptional and translational controls. Biol Rev. 2004;79:207–33. 15005178 10.1017/s1464793103006195

[80] Protas M, Jeffery WR. Evolution and development in cave animals: from fish to crustaceans. WIREs Dev Biol. 2012;1:823–45. 10.1002/wdev.61 PMC3620605 23580903

[81] Kowalko JE, Ma L, Jeffery WR Genome editing in *Astyanax mexicanus* using transcription activator-like effector nucleases (TALENs). J Vis Exp. 2016;112:e54113. 10.3791/54113 PMC4993240 27404092

[82] Rodriguez-Morales R, Gonzalez-Lerma P, Yuiska A, Han JH, Guerra Y, Crisostomo L, et al. Convergence on reduced aggression through shared behavioral traits in multiple populations of *Astyanax mexicanus*. BMC Ecol Evol. 2022;22:116. 36241984 10.1186/s12862-022-02069-8 PMC9563175

[83] Duboué ER, Keene AC, Borowsky RL. Evolutionary convergence on sleep loss in cavefish populations. Curr Biol. 2011;21:671–6. 21474315 10.1016/j.cub.2011.03.020

[84] Ma L, Zhao Y Cavefish of China. In Encyclopedia of caves*.* Elsevier; 2012. pp. 107–125.

[85] Chen B, Mao T, Liu Y, Dai W, Li X, Rajput AP, et al. Sensory evolution in a cavefish radiation: patterns of neuromast distribution and associated behaviour in *Sinocyclocheilus* (Cypriniformes: Cyprinidae). Proc R Soc B Biol Sci. 2022;289:20221641. 10.1098/rspb.2022.1641 PMC9554722 36476002

